# Urinary Markers for Bladder Cancer Diagnosis and Monitoring

**DOI:** 10.3389/fcell.2022.892067

**Published:** 2022-05-02

**Authors:** Seung-Hwan Jeong, Ja Hyeon Ku

**Affiliations:** ^1^ Department of Urology, Seoul National University Hospital, Seoul, South Korea; ^2^ Department of Urology, Seoul National University College of Medicine, Seoul, South Korea

**Keywords:** bladder cancer, urine marker, diagnosis, Hematuria, screening

## Abstract

Hematuria is a typical symptom of bladder cancer which enables early detection of bladder cancer. However, reliable diagnostic tools for bladder cancer using urine samples or other non-invasive methods are lacking. Tremendous attempts have been tried and revealed fancy works to convey definitive diagnostic power using urine samples. In this paper, we reviewed urinary markers for bladder cancer and compared their efficacies.

## Introduction

Bladder cancer is the 6^th^ most common cancer in men and 17th most common cancer in women. The incidence of bladder cancer is relatively high in developed countries, and because of rapid industrialization, its worldwide incidence is increasing (Saginala et al., 2020). As bladder cancer results in gross or microscopic hematuria, approximately 80% of bladder cancers are diagnosed as non-muscle invasive bladder cancer (NMIBC) (Zhu et al., 2019). However, the recurrence rate of NMIBC is as high as 60% within 1 year of the first diagnosis (Mancini et al., 2020). The gold standard for the confirmative diagnosis of bladder cancer is cystoscopic examination, but its invasiveness hinders its early utilization requiring non-invasive diagnostic marker (Zhu et al., 2019). The bladder is a hollow organ that preserves urine; thus, tremendous attempts have been made to facilitate non-invasive diagnostic tools using urine for bladder cancer. Nevertheless, there have been limitations to these attempts due to the restricted efficacy reflecting the current status, which warrants skipping further cystoscopic examinations. In this review, we summarize the representative tests for bladder cancer using urine and suggest future directions.

## Urine Cytology

Urine cytology examines the morphological changes in exfoliated cells from the urinary tract to assess abnormalities (Woldu et al., 2017). The sensitivity of urine cytology varies with cancer grade. In high-grade urothelial cancer, the sensitivity is as high as 86%, but 20–50% in low-grade cancers (Zhu et al., 2019). Furthermore, urine cytology suffers from subjective results upon examination and variables related to low cellularity, infections, and artifacts. The Paris Working Group released a standardized reporting system for urine cytology to improve the objectivity of results (Barkan et al., 2016). To yield more cellularity, catheterization and washing methods can be attempted in some situations, but are limited because of the invasiveness and artifacts caused by the maneuvers (Sullivan et al., 2010). However, the specificity of urine cytology is 90–100%, empowering its diagnostic value in addition to cystoscopy in high-risk bladder cancer. In bladder cancer patients managed with transurethral resection, urine cytology and cystoscopy examinations are recommended every 3–6 months by the National Comprehensive Cancer Network guidelines (Flaig et al., 2020). Abnormal urine cytology results imply the presence of a tumor, but negative results do not ensure normal conditions.

## Overview of Urinary Molecular Markers

### Molecular Markers

Considering that the purpose of urine testing is to avoid unnecessary cystoscopic examinations, a high negative predictive value is required for molecular marker tests. Unfortunately, the reported markers only provided higher sensitivity with lower specificity compared to urine cytology, hampering their negative predictive value. Thus, none of them is in use with recommendations from the guidelines.

### Nuclear Matrix Protein-22

Borderline results from urine cytology, such as atypical cells, are confusing for follow-up and diagnosis. Nuclear matrix protein-22 (NMP-22) mediates the appropriate distribution of chromatin in cellular proliferation and exists at a low level in normal cells but at a level as high as 25 fold in tumorous conditions (Têtu, 2009). NMP-22 improves the positive predictive value of urine cytology from 30 to 60% (Ahn et al., 2011). The NMP-22 Bladder Cancer ELISA Test Kit quantifies the level of NMP-22 in urine to provide a sensitivity of 50–70% and a specificity of 60–90% for cancer detection. However, the variable results between individuals and institutions restrict their use in clinical settings (Murakami et al., 2021). NMP22 BladderChek delivers an easy and direct result within 30 min at the point-of-care, with a sensitivity of 56% and specificity of 88%. These values are especially higher in more advanced-stage cancers. The pooled positive and negative likelihood ratio were 4.36 and 0.51, respectively (Wang et al., 2017). Thus, NMP22 BladderChek can be used in high-risk patients but has limited clinical applications.

### Bladder Tumor Antigen (BTA), BTAstat and BTA-TRAK

The bladder tumor antigen (BTA) assay detects complement factor H-related protein released from bladder cancer. BTA stat is a point-of-care form, and BTA-TRAK is an ELISA kit that shares similar sensitivity and specificity of 58 and 73%, respectively (Têtu, 2009; Villicana et al., 2009). BTA analysis is approved by the FDA for monitoring bladder cancer with cystoscopy, but not for initial screening.

### UroVysion in Fluorescence *in situ* Hybridization

Bladder cancer exhibits aneuploidy of chromosomes (3, 7, and 17) and deletion of the 9p21 locus. UroVysion uses fluorescence *in situ* hybridization (FISH) to detect chromosomal abnormalities (Villicana et al., 2009). The sensitivity of UroVysion varies depending on the disease status from low to high T stage and tumor grade. The overall sensitivity was approximately 72% and the specificity was 83%, providing a higher diagnostic AUC of 0.867 compared with 0.626 for urine cytology (Villicana et al., 2009). Because of the complicated procedures required by cytopathology experts and expensive equipment, the expansion of this method is restricted.

### Urine miRNA

MicroRNAs (miRNAs) are small non-coding RNAs consisting of 20–22 nucleotides, which regulate protein expression through post-transcriptional gene regulation via RNA silencing. The miRNA is transcribed in the nucleus by RNA polymerase II, reading the long primary transcript, and matured by Drosha and Dicer to form a single-stranded structure pairing with the 3’ untranslated region of messenger RNAs (mRNAs) (Kuehbacher et al., 2007). miRNAs are relatively stable in body fluids, including blood and urine, compared to mRNA, which allows miRNAs to be an appropriate diagnostic target for bladder cancer. Although the alteration of miRNA has not been fully elucidated in the bladder, miRNA is dysregulated in bladder cancer to promote proliferation and progression through epithelial to mesenchymal transition and inhibit apoptosis (Enokida et al., 2016; Hofbauer et al., 2018). Hofbauer et al. reported a diagnostic model using six miRNAs (let-7c, miR-135a, miR-135b, miR-148a, miR-204, miR-345) to provide a diagnostic AUC of 88.3% (Hofbauer et al., 2018). In a meta-analysis of urine miRNA for bladder cancer detection, a combination test with multiple miRNAs was found to be superior to the single miRNA test (Kutwin et al., 2018). Urinary miRNAs have implications not only for diagnosis, but also for prognosis. For an instance, miR-9, miR-182, and miR-200b have been associated with muscle invasiveness and poor prognosis (Braicu et al., 2015). Huang et al. reported that miR-125b acts as a tumor suppressor and is downregulated in bladder cancer (Ahn et al., 2011). Wang et al. found that the urinary miR-200 family, miR-192, and miR-155 are downregulated in bladder cancer compared with controls (Ahn et al., 2011). The ratio of urinary miR-126 to miR-152 is elevated in bladder cancer, with a sensitivity and specificity of 72 and 82%, respectively (Hanke et al., 2010). Otherwise miR-126, miR-96 show similar sensitivities and specificity of 71–72% and 82–89% (Enokida et al., 2016). The six-miRNA panel of miR-152, miR-148b-3p, miR-3187-3p, miR-15b-5p, miR-27a-3p, and miR-30a-5p had a high diagnostic yield, represented by an AUC of 0.899. Furthermore, high levels of miR-152 and miR-3187-3p were associated with poor recurrence-free survival in NMIBC (Jiang et al., 2015).

### Urine Cell-free DNA

Urine cell-free DNA (cfDNA) originates from several sources, including urothelial cells, transrenal circulating DNA, and bacteria (Tse et al., 2021). The majority of urine cfDNAs is from urothelial cells lining the urinary tract, which can be shed off and undergo necrosis or apoptosis to release DNA from the cells. Unlike normal cells, tumor cells release longer DNA segments with higher integrity (Casadio et al., 2013). Thus, a higher proportion of cfDNA to cellular DNA reflects the presence of tumor cells (Ou et al., 2020). Detection of urine cfDNA integrity and mutations is available for assessing bladder cancer. The integrity of urine cfDNA is much higher in bladder cancer than under normal conditions (Brisuda et al., 2016). Furthermore, the length of DNA fragments is relatively longer in bladder cancer, implying that it originates from the necrotic debris of cancer cells (Tse et al., 2021). Urine cfDNA tests can detect bladder cancer with a sensitivity of 57–86% and specificity of 72–84% (Salvi et al., 2016). The amount of urine cfDNA depends on the volume and concentration of the urine. Thus, urine creatinine-adjusted DNA concentrations can be used for normalization. Notably, urine cfDNA of 400-bp was much more abundant than that of the control, whereas the median concentration was only higher by 1.5 fold in bladder cancers (Tse et al., 2021). Additionally, a urine cfDNA concentration over 250 ng/ml was indicated as the threshold value to predict bladder cancers (Tse et al., 2021). Urine cfDNA sequencing has revealed valuable genetic mutations for the detection of bladder cancer. For an instance, the frequently detected mutations in bladder cancers such as TERT, FGFR3, TP53, PIK3CA, and KRAS were significantly altered in urine cfDNAs showing cancer detection rate using these five gene panel with a AUC confidence interval of 0.94 (Ou et al., 2020). Telomerase reverse transcriptase (TERT) mutations are observed in 60–85% of bladder cancers with frequently mutated promoter regions C228T and C250T (Avogbe et al., 2019). TERT promoter mutations in urinary cell-free and cellular DNA can be detected in urothelial cancer with a sensitivity of 86%, up to 93.9% when combined with urine cytology, and with a specificity of 94.7%. The fibroblast growth factor receptor3 (FGFR3) mutation is one of the most commonly detected mutations in bladder cancer, occurring in approximately 12% of all cases and in 70% of low-grade NMIBC (Zuiverloon et al., 2010; Weinstein et al., 2014). Urinary FGFR3 mutation analysis has a sensitivity of 73% and a specificity of 87%, with positive results implying shorter recurrence periods (Ahn et al., 2011). In another study, urine cfDNA for droplet digital polymerase chain reaction of the TERT promoter and FGFR3 provided a sensitivity of 68.9% and specificity of 100%, with an enhanced sensitivity of 85.9% when combined with urine cytology (Hayashi et al., 2020). Moreover, patients with TERT mutations in urine cfDNA showed worse prognosis compared with negative patients.

Tumors shed off DNA and the mutations harbor distinct alterations of DNA sequences according to tumor type and development, but the sensitivity of the cfDNA test is relatively low, making it a more appropriate method with improvements. DNA methylation is highly preserved throughout species and organs, which vary in tumor cells, implying its utilization for cancer detection (Lee et al., 2020). The detection of cfDNA mutations targeting single nucleotide variants and copy number alterations has caveats due to confounding signals of white blood cells and clonal hematopoiesis of indeterminate potential. Methylation sequencing of cfDNA surpasses targeted or whole-genome sequencing in the Circulating Cell-free Genome Atlas study (Liu et al., 2020). Epigenetic changes in urine cfDNA have diagnostic value for urothelial cancers. Anouk et al. reported that DNA methylation of urine samples and tumor tissues is significantly correlated, which allows the utilization of urine DNA methylation analysis for the diagnosis of bladder cancer. Among the nine genes reported in their previous study to be associated with bladder cancer according to the methylation status, the GHSR/MAL panel achieved a significant value with an AUC of 0.87 (95% CI, 0.73–1.00) (Hentschel et al., 2020). Yu et al. demonstrated that bladder cancer patients harbor methylation of 11 genes, including SALL3, CFTR, ABCC6, HPR1, RASSF1A, MT1A, ALX4, CDH13, RPRM, MINT1, and BRCA1, in urine samples. Bladder EpiCHeck detects DNA methylation in urine with a panel designed with 15 markers to diagnose bladder cancer with a sensitivity of 68.2% and a specificity of 88.0% (Witjes et al., 2018; Chen et al., 2020). A 2-marker based methylation assay, utMeMA, revealed a superior detection rate in early stage bladder cancer, with a better association with tumor burden (Chen et al., 2020). Furthermore, DNA methylation of urine samples is useful for detecting the recurrence of bladder cancer. Notably, TWIST and NID2 methylation are associated with bladder cancer recurrence with 84 and 96% sensitivity and specificity, respectively. In addition, another study reported that methylation of APC, RASSF1A, and CDK2AP2 is associated with bladder cancer recurrence with a sensitivity and specificity of 87 and 100%, respectively (Kandimalla et al., 2013). Further investigation is required to provide concrete evidence for the clinical use of these examinations.

## Discussion/Conclusion

In bladder cancer, the diagnostic utilization of urine has enormous potential because cancer cells shed materials directly into the urine. Nonetheless, no other urine tests, except for urine cytology, are recommended for the initial diagnosis or follow-up of bladder cancer because of their low sensitivity and specificity. In addition to traditional urinary marker tests, miRNA and cfDNA tests have been investigated and have shown promising results. Next-generation sequencing has enabled deeper analysis of molecular markers in urine, and comprehensive analysis can be achieved in accordance with artificial intelligence to deduce the fundamental assembly of molecular markers. In this regard, further studies are expected to reveal key molecular panels that facilitate accurate diagnosis and reduce invasive procedures.
